# Quantitative detection of dengue serotypes using a smartphone-connected handheld lab-on-chip platform

**DOI:** 10.3389/fbioe.2022.892853

**Published:** 2022-09-15

**Authors:** Nicolas Moser, Ling-Shan Yu, Jesus Rodriguez Manzano, Kenny Malpartida-Cardenas, Anselm Au, Paul Arkell, Chiara Cicatiello, Ahmad Moniri, Luca Miglietta, Wen-Hung Wang, Sheng Fan Wang, Alison Holmes, Yen-Hsu Chen, Pantelis Georgiou

**Affiliations:** ^1^ Centre for Bio-Inspired Technology, Department of Electrical and Electronic Engineering, Faculty of Engineering, Imperial College London, London, United Kingdom; ^2^ Institute of Biopharmaceutical Sciences, College of Medicine, National Sun Yat-Sen University, Kaohsiung, Taiwan; ^3^ Department of Infectious Disease, Faculty of Medicine, Imperial College London, London, United Kingdom; ^4^ School of Medicine, College of Medicine, National Sun Yat-sen University, Kaohsiung, Taiwan; ^5^ Center for Tropical Medicine and Infectious Disease, Kaohsiung Medical University, Kaohsiung, Taiwan; ^6^ Department of Medical Laboratory Science and Biotechnology, Kaohsiung Medical University, Kaohsiung, Taiwan; ^7^ Institute of Systems, Molecular and Integrative Biology, University of Liverpool, Liverpool, United Kingdom

**Keywords:** diagnostics, digital diagnostics, lab-on-chip, point-of-care, nucleic acid, dengue, surveillance, molecular assay

## Abstract

Dengue is one of the most prevalent infectious diseases in the world. Rapid, accurate and scalable diagnostics are key to patient management and epidemiological surveillance of the dengue virus (DENV), however current technologies do not match required clinical sensitivity and specificity or rely on large laboratory equipment. In this work, we report the translation of our smartphone-connected handheld Lab-on-Chip (LoC) platform for the quantitative detection of two dengue serotypes. At its core, the approach relies on the combination of Complementary Metal-Oxide-Semiconductor (CMOS) microchip technology to integrate an array of 78 × 56 potentiometric sensors, and a label-free reverse-transcriptase loop mediated isothermal amplification (RT-LAMP) assay. The platform communicates to a smartphone app which synchronises results in real time with a secure cloud server hosted by Amazon Web Services (AWS) for epidemiological surveillance. The assay on our LoC platform (RT-eLAMP) was shown to match performance on a gold-standard fluorescence-based real-time instrument (RT-qLAMP) with synthetic DENV-1 and DENV-2 RNA and extracted RNA from 9 DENV-2 clinical isolates, achieving quantitative detection in under 15 min. To validate the portability of the platform and the geo-tagging capabilities, we led our study in the laboratories at Imperial College London, UK, and Kaohsiung Medical Hospital, Taiwan. This approach carries high potential for application in low resource settings at the point of care (PoC).

## 1 Introduction

Dengue is a viral disease vectored by *Aedes* mosquitoes which causes up to 400 million infections each year globally. There are four serotypes of dengue virus which can infect humans (DENV 1–4). Although they may circulate together, regional seasonal outbreaks are often attributed to a predominant serotype (most frequently DENV-1 or DENV-2), which may be superseded by another in later years (Guo et al., 2013). Individuals in endemic regions may therefore be exposed to several serotypes during their lifetime. A first infection (“primary dengue”) can cause a range of symptoms which vary depending on the stage and severity of infection. Many of these present with non-specific features, which can be indistinguishable from other causes of acute febrile illness (Guzman et al., 2016). In non-severe DENV infection, these usually resolve after 2–7 days. If the same individual is later infected with a second serotype (“secondary dengue”), this can lead to an enhanced immune reaction, which is more frequently associated with severe disease and death. Features of severe dengue usually occur between day 4–6 of illness. Early, active, supportive treatment is key in the clinical management of severe DENV infection (Word Health Organisation, 2009; Low et al., 2017).

Diagnosis of dengue based on clinical features is challenging, and should be confirmed with a diagnostic test wherever possible (Muller et al., 2017). There is a huge global need for rapid, accurate, portable and low-cost testing for dengue (Peeling et al., 2010). Assays which can quantify viraemia and those which perform well in the context of both primary and secondary dengue are a priority, in order to optimally support the clinical management of patients presenting with febrile illness in remote areas (Katzelnick et al., 2017). Those which can differentiate serotype and perform automatic case-notification will be crucial in improving global surveillance, reporting live epidemiological data to decision makers, allowing for forthcoming prospective observational studies of dengue and informing clinical trials of vaccine and therapeutics (Peeling et al., 2010; Rodriguez-Manzano et al., 2018). Existing methods described in Section S1 suffer from a lack of integration with electronic health records to enable surveillance, or a reliance on high level laboratories which are often not available in low-resource settings of low-and middle-income countries.

When trying to match the analytical performance of lab-based Nucleic Acid Amplification Tests (NAATs) point-of-care diagnostics (PoC), significant challenge lies with miniaturisation and reliability of the portable solution. New technologies have started to emerge which use innovative solutions involving smartphones as a tool now available to a wide range of the population, however they often still rely on optical instrumentation which is bulky and expensive (Chen et al., 2017; Priye et al., 2017). The step to achieving handheld detection may be taken when considering label-free detection methods which do not need optical equipment. Electrochemical sensing has demonstrated opportunities for detecting charged molecules reliably and rapidly in small volumes (Goral et al., 2006; Kurbanoglu et al., 2016). Among these sensors, the Ion-Sensitive Field-Effect Transistor (ISFET) has appeared as an ideal candidate as it may be fabricated using a similar structure to the Metal-Oxide-Semiconductor Transistor (MOSFET) which is the building block of Complementary Metal-Oxide-Semiconductor (CMOS) technology (Moser et al., 2016). Not only has CMOS technology revolutionised modern electronics by offering low-power, low-cost and scalable architectures following Moore’s law (Schaller, 1997), it has also enabled the next generation of affordable full genome sequencing using ISFETs, unlocking huge potential towards biomedical applications (Rothberg et al., 2011; Hayden, 2014). Applied to diagnostics, it offers the potential to achieve scalable, rapid, low-cost and robust dengue detection tests. Paired with mobile technologies such as smartphones, this innovative approach bridges the gap towards digitalisation of diagnostics, enabling testing outside of the hospital and ultimately democratising diagnostics (Fleming et al., 2021).

We have previously reported molecular detection using a lab-based CMOS Lab-on-Chip (LoC) platform coupled with loop-mediated isothermal amplification (LAMP), an isothermal reaction where nucleotide incorporation induces a release of protons during amplification, monitored in real time by thousands of ISFETs on a chip (Moser et al., 2018; Malpartida-Cardenas et al., 2019). We have implemented this approach with a handheld LoC platform for molecular detection at the PoC called Lacewing and demonstrated performance for infectious diseases such as COVID-19 (Rodriguez-Manzano et al., 2021), fungal diseases including *Aspergillus Fumigatus* (Yu et al., 2019) and antimicrobial resistance with *mcr-9*, a strain of colistin resistance (Rodriguez-Manzano et al., 2020).

In this paper, we demonstrate for the first time the combination of rapid detection of dengue on a LoC platform (*Lacewing*) and integration with a smartphone app (*Firefly*) designed for ease of use and data connectivity. This step is essential to consider the technology as digital diagnostics and address challenges such as data bandwidth and connectivity structure between the device and the cloud. We show that quantitative detection using a real-time reverse-transcriptase (RT-LAMP) assay is achieved in under 30 min, and that the results obtained on a real-time benchtop instrument (RT-qLAMP) match the performance of the assay on the LoC device (RT-eLAMP) for DENV-1 and DENV-2. Further to the validation at Imperial College London (UK), we provide results from a clinical validation at Kaohsiung Medical Hospital (Taiwan) with 9 DENV-2 clinical samples and show good correlation between RT-qLAMP and RT-eLAMP. This exercise allows for a demonstration of the capabilities for real-time syncing of diagnostic data in the cloud for dengue surveillance.

## 2 Materials and methods

### 2.1 Synthetic RNA and clinical samples

Synthetic RNA was transcribed from synthetic DNA (gBlock) obtained from Integrated DNA Technologies (IDT). The synthetic DNA was resuspended in TE at 5 ng/μL and transcribed into RNA using HiScribe T7 High Yield RNA Synthesis Kit (New England Biolabs) following manufacturer’s protocol. The RNA product was then purified using the RNAqueous total RNA isolation kit from Thermofisher following manufacturer’s protocol.

Serum samples were collected and processed by the Microbiology laboratory at a Kaohsiung Medical University (Taiwan). A total of 9 serum samples were extracted using the QIAamp Viral RNA Mini Kit following manufacturer’s protocol for LAMP experiments. All samples were characterized by several methods as shown in Table 1 including: 1) the MULTISURE Dengue Ab/Ag Rapid Test from MP Diagnostics that detects IgA, IgM, IgG antibodies and NS1 dengue antigen in human serum, and 2) viral culture which was performed by infecting C6/36 cells (*Aedes albopictus*; ATCC CRL-1660) with dengue virus from patients’ serum. C6/36 cells were grown in minimum essential medium (MEM, Sigma) which was supplemented with 10% fetal bovine serum and non-essential amino acids at 28^*o*^C in 5% CO_2_. The presence of DENV was determined by immunofluorescence at day 5 after inoculation.

**TABLE 1:** Clinical samples characterization for the 9 serum samples run with RT-qLAMP in the real-time PCR instrument, RT-eLAMP on LACEWING, Ab/Ag rapid test and viral culture.

Sample ID	Serotype	RT-qLAMP	RT-eLAMP	MULTISURE rapid test^†^				Viral culture^‡^
				IgG	IgM	IgA	NS1	
1	DENV2	+	+	—	—	—	+	+
2	DENV2	+	+	—	—	—	+	+
3	DENV2	+	+	—	+	—	—	+
4	DENV2	+	+	—	—	—	+	+
5	DENV2	+	+	—	—	—	+	+
6	DENV2	+	+	—	—	—	+	+
7	DENV2	+	+	—	—	—	+	+
8	DENV2	+	+	—	—	—	+	+
9	DENV2	+	+	—	—	—	+	+

### 2.2 LAMP primers and amplification reaction conditions

LAMP primer sequences used in this study were previously reported by (Hu et al., 2015). Primers were synthesized by IDT and resuspended in TE buffer at 100 µM. A 10X primer mixture was prepared as follows: 20 µM BIP/FIP, 10 µM LF and LB, and 2.5 µM B3/F3.

#### 2.2.1 RT-qLAMP reaction

Experiments were done with 4 repeats and each LAMP reaction contained the following: 1 µL 10X custom isothermal buffer, 0.6 µL MgSO4 (stock at 100 mM), 1.4 µL dNTPs (stock at 10 mM each), 0.5 µL BSA (20 mg/ml), 1.60 µL Betaine (5 M), 0.25 µL SYTO 9 Green (20 µM stock), 0.40 µL of Bst 2.0 DNA Polymerase (8,000 U/µL) (New England Biolabs, UK), 0.25 µL of AMV Reverse Transcriptase (25 U/µL) (Promega Corporation, US), 0.1 µL of RNase inhibitor (stock at 20 U/µL), 1 µL of 10X primer mixture, 1 µL of genomic RNA (clinical samples) or synthetic RNA template solution ranging from 10 to 10^6^ copies/µL, and enough nuclease-free water to bring the volume to 10 µL. The RT-qLAMP reactions were performed in a LightCycler 96 Real-Time PCR system (LC96) (Roche Diagnostics) at 63^*o*^C for 25 min. Quantification metric is based on the *C*_*t*_ method setting the threshold at 0.1 after normalization.

#### 2.2.2 RT-eLAMP reaction

Experiments were done in triplicates and each LAMP reaction contained the following: 1 µL 10X custom isothermal buffer, 0.6 µL MgSO4 (stock at 100 mM), 0.56 µL dNTPs (stock at 25 mM each), 0.5 µL BSA (20 mg/ml), 1.6 µL Betaine (5 M), 0.04 µL of Bst 2.0 DNA Polymerase (120,000 U/µL) (New England Biolabs, UK), 0.23 µL of NaOH (stock at 0.2 M), 0.25 µL of AMV Reverse Transcriptase (25 U/µL) (Promega Corporation, US), 0.10 L of RNase inhibitor (20 U/µL stock) (Thermo Fisher Scientific), 1 µL of 10X primer mixture, 1 µL of genomic RNA (clinical samples) or synthetic RNA template ranging from 10 to 10^6^ copies/µL, and enough nuclease-free water to bring the volume to 10 µL. The RT-eLAMP reactions were performed on the LoC platform at 63^*o*^C for 25 min.

### 2.3 ISFET-based potentiometric sensing

**FIGURE 1:**
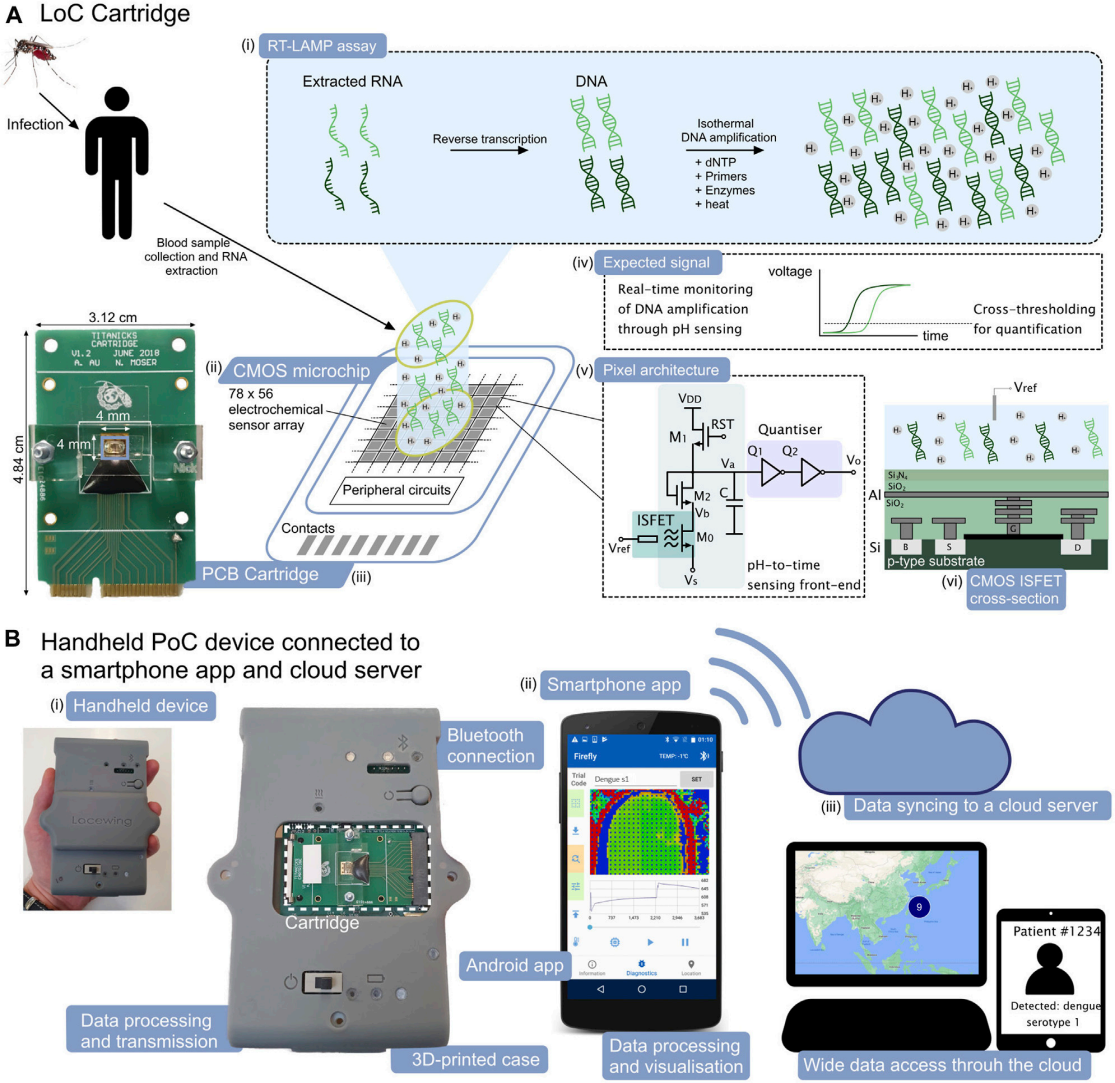
ISFET-based LoC platform and smartphone application for dengue molecular diagnostics. (A). LoC cartridge for nucleic acid amplification and detection on-chip. (i) Schematic representation of the RT-LAMP assay, (ii) Schematic view of the CMOS microchip with 78 × 56 integrated electrochemical sensors, (iii) Picture of the PCB cartridge with microchip encapsulated using epoxy and acrylic manifold, (iv) Expected form of the electrochemical signal associated with the nucleic acid amplification, (v) CMOS pixel architecture with time encoding of the signal and (vi) cross section of the ISFET sensor in unmodified CMOS technology. **(B).** Handheld PoC “Lacewing” device and back-end smartphone interface. (i) Photographic view of the handheld device for data processing and transmission *via* Bluetooth, (ii) Layout of the smartphone app and (iii) Concept view of the data syncinc to the cloud server and data visualisation.

ISFETs are solid-state potentiometric sensors with a structure similar to the MOSFET, where the gate oxide is replaced with an insulating layer and the gate voltage is applied through a reference electrode (typically Ag/AgCl) in contact with the solution (Bergveld, 2003). The potential of this sensor became evident when its implementation in unmodified CMOS technology was reported by extending the floating gate of the device to the top layer of the metal stack as shown in Figure 1A (Bausells et al., 1999). The native surface passivation layer (typically Si_3_N_4_ or SiO_2_) is used as the sensing layer, which selectively binds protons at its surface and therefore provides inherent pH sensitivity. A change in pH modulates the threshold voltage of the device compared to its MOSFET equivalent following the relation

$$V_{th\left(ISFET\right)}=V_{th\left(MOSFET\right)}+\gamma +\alpha S_{N}\;pH$$ (1)

where *γ* is a constant chemical term non dependent of pH, *S*_*N*_ is the ideal or Nernstian sensivity approximately equal to 59 mV/pH at ambient temperature and *α* is the deviation from this ideal sensitivity due to the double layer capacitance. The chemical contribution of the threshold voltage is often denoted as *V*_*chem*_ (Georgiou and Toumazou, 2009).

$$V_{\mathrm{chem}}=\gamma +\alpha S_{N}\;Vchem$$ (2)

Due to their implementation in commercial technology, ISFETs suffer from non-idealities compromising the reliability and dynamic range of the readout. Firstly, charge trapped at the floating gate and the passivation layers may induce an offset in the threshold voltage of the device. Without compensation, the offset forces a wide dynamic range for the on-chip quantiser, significantly increasing power consumption and area for the system. Secondly, surface effects associated with hydration cause a slow temporal drift in the ISFET output, which may compromise the detection of slow chemical processes (Moser et al., 2016). Overall, the ISFET threshold voltage can be expressed as

$$V_{th\left(ISFET\right)}=V_{th\left(MOSFET\right)}+V_{\mathrm{chem}}+V_{tc}+V_{\mathrm{drift}}$$ (3)

where *V*_*tc*_ is the offset voltage associated with trapped charge and *V*_*drift*_ is the voltage associated with drift.

In this work, offset is compensated on-chip while drift is alleviated by (1) conditioning the sensor in solution for several hours before running the experiment and 2) implementing a compensation algorithm based on characteristics of the DNA amplification curve.

### 2.4 On-chip pH-LAMP detection

The label-free detection mechanism relies on the release of protons with successive nucleotide incorporation during nucleic acid amplification, decreasing the pH in solution and generating a local signal sensed by the ISFET (Moser et al., 2018; Malpartida-Cardenas et al., 2019).

### 2.5 Handheld LoC device

The *Protonic* LoC cartridge shown in Figure 1A builds upon our previous architecture of encapsulated CMOS microchip on a printed circuit board (PCB) (Moser et al., 2018). The 4 × 4 mm microchip integrates an array of 78 × 56 ISFET pixels to perform electrochemical sensing of the RT-eLAMP reaction.

The CMOS microchip design is the first mixed-signal ISFET array, integrating in each pixel an analogue sensing front-end, a digital quantiser and a 10-bit static random access memory (SRAM). The change in threshold voltage associated with pH variation is converted into current through the ISFET M0 and then in the time domain with the inverters Q1-2, providing improved immunity to interference during transmission (Figure 1A). The memory array stores a voltage compensation value as part of an initial offset calibration stage, allowing the sensors to be initialised at a baseline frame at the start of the amplification reaction. The system relies on a finite state machine (FSM) for sensor calibration and data transmission. Due to the use of standard CMOS technology which is characterised by a thick passivation layer and a non-ideal sensing surface, our previous results demonstrated an attenuated sensitivity of 13 mV/pH. The use of time domain encoding circuits has allowed to demonstrate an optimal measured resolution of 0.019 pH (Moser et al., 2018).

The main device shown in Figure 1B relies on a PCB with a microcontroller to perform data acquisition from the cartridge and implement the sensor calibration algorithm. Furthermore, the microcontroller performs closed-loop temperature regulation using on-chip real-time sensors scattered throughout the array and a Peltier module in contact with the bottom side of the cartridge. The regulation is programmed to maintain a temperature of 63^*o*^C for the LAMP reaction (Moser et al., 2018). Lastly, the data is transmitted to the smartphone *via* Bluetooth.

### 2.6 Data processing

Data analysis is performed by averaging the time series from each active sensor and compensating for sensor drift. The algorithm extracts the signal associated with DNA amplification by identifying the maximum derivative of the sensor output and performing an exponential fitting (Malpartida-Cardenas et al., 2019; Rodriguez-Manzano et al., 2020, 2021). Quantification metric is based on the *C*_*t*_ method where the threshold is set to 0.1 and the reference time *t* = 0 is when the reaction chamber has settled to the temperature of 63^*o*^C.

### 2.7 Android-based smartphone app

**FIGURE 2:**
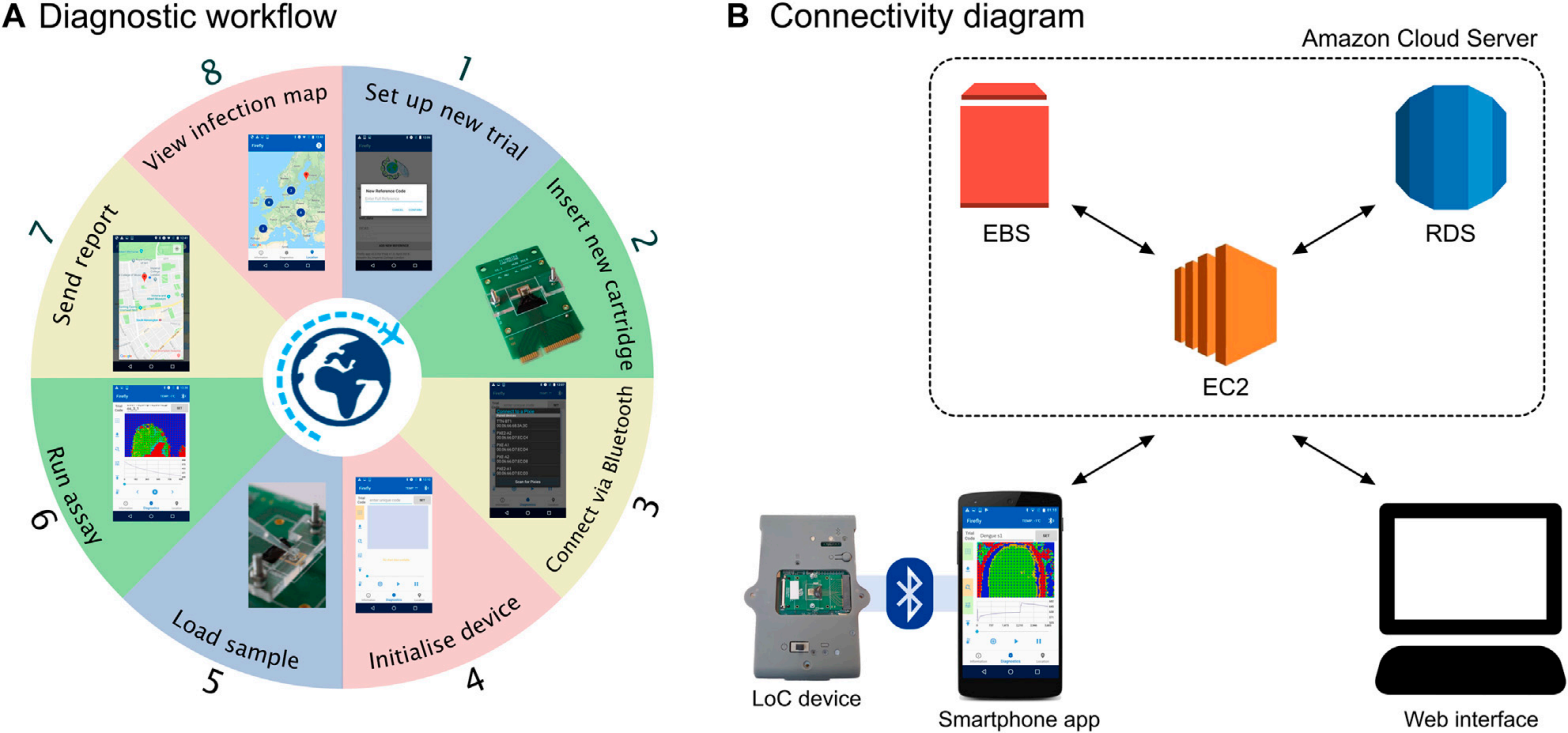
Overview of the smartphone setup. **(A)** Diagnostic workflow involving the Lacewing platform and the Firefly app. **(B)** Diagram of the connectivity structure between the Firefly app, the AWS cloud server and the web interface.

We leveraged the use of smartphones as a device widely available to users around the world to interface with our new PoC test aimed at remote settings. We designed an Android app to interface to the LoC platform and provide diagnostic results. The app allows to acquire the time series from the CMOS sensors in bytes via Bluetooth, visualise the output in real-time, process the data, synchronise the results to a cloud server and geo-tag the location of testing. The overall diagnostic workflow using the device and the app is depicted in Figure 2A.

The app was designed using Android Studio v3.0 IDE and Gradle v4.1 compiler-builder for Java code for all devices. Although our experimental setup used a Nexus 5 phone running Android v6.0.1 Marshmallow, the app supports all OS versions above Android v4.0.3 Ice Cream Sandwich, which includes virtually all Android devices currently available in the market. 2D data visualisation was achieved with minimal RAM requirements by generating and displaying a PNG image of each frame with data-to-colour conversion.

### 2.8 Secure cloud server and web interface

The server was set up using Amazon Web Services (AWS) as this offered a simple solution for cloud storage and access based in the UK where this study was initiated. The back-end system was built on AWS Virtual Private Cloud (VPC).

The server was designed following the structure of Figure 2B and based on three components:

1) The Elastic Compute Cloud (EC2) is a web service acting as a cloud computing unit, handling computing, query processing and connection to the Android smartphone and web interface.
2) The Elastic Block Store (EBS) is the server storage for the EC2.
3) The Relational Database Service (RDS) is the cloud database based on a MySQL engine.

The web interface was designed to access data files and provide a view of all experiments on an embedded Google Maps tool.

### 2.9 Statistical analysis

To evaluate correlation between RT-qLAMP and RT-eLAMP results, statistical significance of the results was assessed using p-values calculated by Student’s t-test with a two-sided distribution. A p-value > 0.05 was considered to show lack of statistical significance.

## 3 Results and discussion

### 3.1 RT-qLAMP assay

**FIGURE 3:**
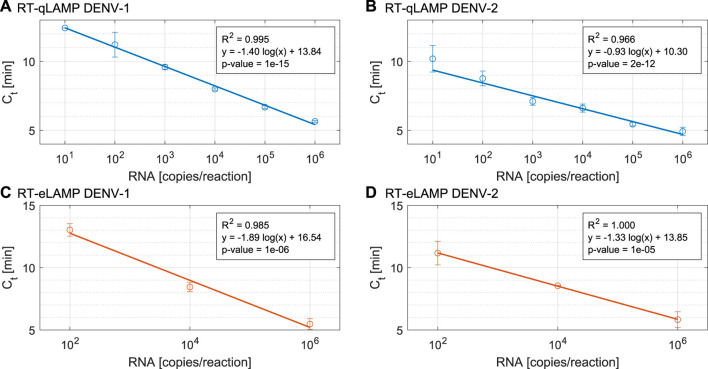
Performance of the DENV RT-qLAMP assay (n = 4) for **(A).** DENV-1 and **(B).** DENV-2 and Performance of the DENV RT-eLAMP assay (n = 3) for **(C).** DENV-1 and **(D).** DENV-2.

Analytical sensitivity of our RT-qLAMP assay was evaluated with 10-fold serial dilutions of synthetic RNA for DENV-1 and DENV-2 ranging from 10 to 10^6^ copies/reaction and in triplicates. The standard curves shown in Figures 3A,B achieve a limit of detection (LoD) of 10 copies/reaction (which corresponds to 1.66 a.m.) and demonstrate statistical significance (p-value = 10^–15^, 2 × 10^–12^) and high linear correlation (*R* ^2^ = 0.995, 0.966). Table 2 provides the time-to-positivity (TTP) values and show that they are within 5.6–12.4 min (DENV-1) and 4.6–11.1 min (DENV-2), with all standard deviations below 1 min. Non-template controls (NTCs) were run in triplicates and no indication of non-specific amplification or primer dimer was observed during the 25-min reaction time. Melting curve analysis (Section S2) was carried out to confirm assay specificity. Overall, these results validate the performance of the LAMP assays with our RT-qLAMP protocol.

**TABLE 2:** Performance of the DENV RT-qLAMP (n = 4) and RT-eLAMP (n = 3) assays showing cross-threshold *C*_*t*_ value and associated standard deviation across repeats. For 10^1^ copies/reaction, we indicate the number of positive repeats.

	DENV-1				DENV-2			
	RT-qLAMP		RT-eLAMP		RT-qLAMP		RT-eLAMP	
RNA	C_t_	Std	C_t_	Std	C_t_	Std	C_t_	Std
[Copies/rxn]	[min]	[min]	[min]	[min]	[min]	[min]	[min]	[min]
**10** ^**1**^	12.42	0 (n = 1)	—	—	10.19	0.96 (n = 3)	—	—
**10** ^**2**^	11.21	0.9	13.03	0.5	8.75	0.54	11.17	0.95
**10** ^**3**^	9.58	0.17	—	—	7.1	0.28	—	—
**10** ^**4**^	7.99	0.08	8.44	0.37	6.61	0.3	8.54	0.04
**10** ^**5**^	6.67	0.12	—	—	5.44	0.13	—	—
**10** ^**6**^	5.64	0.06	5.47	0.43	4.92	0.31	5.83	0.63

### 3.2 RT-eLAMP assay

To demonstrate translation of the RT-qLAMP assay to the LoC platform, we ran RT-eLAMP validation on the Lacewing platform at Imperial College London, UK. We tested the DENV-1 and DENV-2 assays for a range of three concentrations of synthetic RNA (10^2^, 10^4^ and 10^6^ copies/reaction) in triplicates. We monitored the sensor output in real-time during temperature regulation for 30 min. After data post-processing and linearisation, the corresponding RT-eLAMP amplification curve extracted from the sensor is directly comparable to RT-qLAMP curve. Figures 3C,D shows the standard curves which demonstrate high linear correlation (*R* ^2^ = 0.985, 1) and statistical significance (p-values = 10^–6^, 10^–5^). The RT-eLAMP assay achieves a similar speed to the RT-qLAMP with TTP values of 5–13.5 min (DENV-1) and 5.3–12 min (DENV-2), and a similar standard deviation between repeats (Table 2). NTCs were run in triplicates and amplification was not detected during the 25-min reaction time.

### 3.3 Clinical validation in Taiwan

We led a study with the Lacewing platform in the laboratory at Kaohsiung Medical Hospital, Taiwan, to test clinical samples from patients infected with DENV-2. The application of the device outside of Imperial College London illustrated that our LoC device is indeed portable and may be applied to new settings without the need for additional equipment.

**FIGURE 4:**
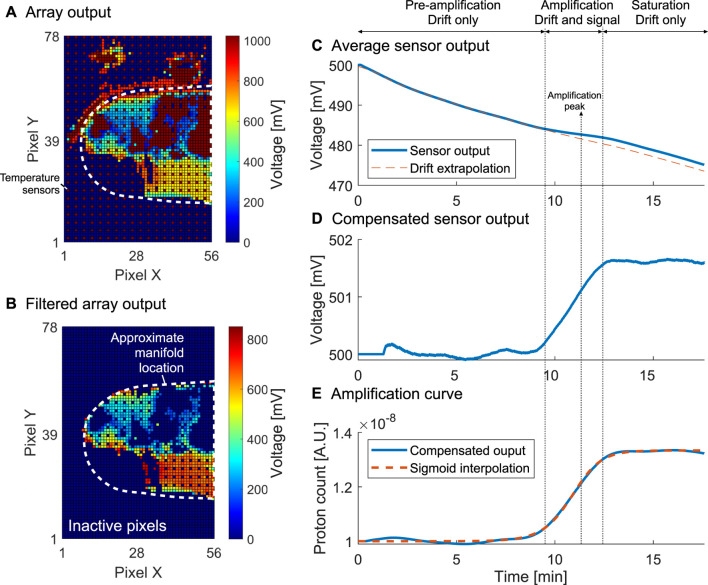
Processing workflow for the electrochemical sensor output illustrated for sample 3. **(A).** Unprocessed initial array output with shown temperature sensors and approximate manifold location. **(B).** Initial array output after filtering out inactive sensors. **(C).** Sensor output averaged over active sensors with drift extrapolation. **(D).** Averaged sensor output with drift compensation. **(E)**. Linearised curve with sigmoidal fitting corresponding to the final amplification curve from which a TTP can be extracted.

The workflow used to extract the outcome from the electrochemical sensor array is illustrated in Figure 4. Figure 4A shows the raw sensor output representing the first “frame” of the dataset. Temperature sensors spread throughout the array and used to perform temperature regulation identified in dark red. An initial filtering step is used to identify sensors which are in the readout range and respond to a variation in the reference electrode voltage *V*_*ref*_. Figure 4B shows the result of the filtering where all inactive pixels have been artificially set to 0 mV. The sensors shown are denoted as active. Figure 4C represents the average output from the active sensors, which corresponds to a times series over the course of the molecular reaction. An amplification peak is identified by computing the max derivative of the curve, and corresponds to an amplification event. The algorithm then identifies three regions of the reaction: 1) pre-amplification where the sensor output is dominated by drift, 2) amplification where the output is the sum of drift and signal associated with the release of protons, and 3) post-amplification or saturation where the output is once again dominated by drift. By interpolating the drift in regions 1) and 3) using an exponential drift model (Jamasb et al., 1998) we obtain the compensated curve of Figure 4D reflecting the voltage change detected in real time. Due to the proportionality of the voltage to pH, the output is then linearised to reflect the nucleic acid amplification in Figure 4E.

**FIGURE 5:**
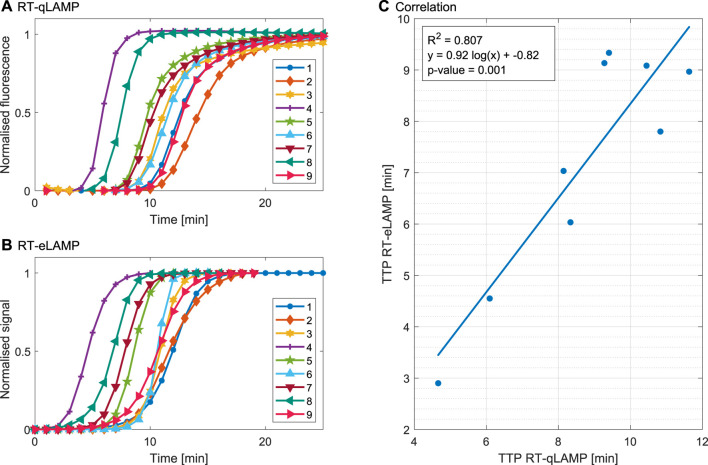
Validation of the RT-LAMP assay for DENV-2 clinical samples in the gold-standard qPCR instrument and the LoC Lacewing platform based on one repeat. **(A)** Amplification curves indicating normalised fluorescence for the RT-qLAMP assay. **(B)** Compensated, linearised and normalised sensor output curves corresponding to amplification curves for the RT-eLAMP assay. **(C)** Correlation between the TTP calculated from *C*_*t*_ RT-qLAMP and RT-eLAMP assays. The results demonstrate good correlation for quantification of the samples.

Figure 5 illustrates the performance of the platform for one repeat with each sample. The post-processed extracted amplification curves from the RT-qLAMP and the RT-eLAMP assays are compared in Figures 5A,B both showing sigmoidal shapes. The speed of the reaction is captured by the two platforms, with samples 4 and 8 leading to the fastest amplifications (resp. 4.7 min/2.9 min and 6.1 min/4.5 min). Discrepancies can be seen in the post-amplification region, where the RT-eLAMP curves reach saturation faster than the RT-qLAMP ones. This effect originates from the drift extrapolation step of the processing workflow, where it is challenging to accurately define when the signal associated with DNA amplification ends. Hence the drift compensation step may cancel contributions from this signal during the end of amplification and artificially induce saturation. However, the essential metric of quantification is the cross-threshold *C*_*t*_ value which solely depends on the start of amplification and is not affected by inaccuracies in the saturation phase.

The measured TTP values of Figure 5C range from 2.8 to 9.5 min and the results demonstrate good correlation (*R* ^2^ = 0.807) and statistical significance (p-value = 0.001), proving the transferability of the assay to clinical samples.

### 3.4 Digital diagnostics for dengue surveillance

This study has paved the way to novel DENV digital diagnostics with serotyping capabilities, ideally suited for clinical management in healthcare settings of remote areas, and providing real-time epidemiological data for global surveillance. This suitability is due to several key enablers. First, the approach does not rely on optical detection like most commercial instruments used in laboratory settings, requiring a large optical source and detector usually fitted as a benchtop instrument. Instead, we have leveraged potentiometric ISFET sensing to detect protons (Moser et al., 2016), limiting the detection setup to a small format LoC cartridge with a low volume reaction of 20 µL. Second, the electrochemical sensing has been implemented using CMOS technology, benefitting from its economies of scale to provide a low cost solution to manufacturing the LoC cartridge (Moser et al., 2018). Additional components including a standard PCB board and an acrylic manifold are similarly inexpensive. Third, the use of LAMP as an isothermal chemistry has provided a highly specific alternative to PCR that does not require thermal cycling, and the RT-qLAMP assay has been designed to allow for pH variation. We have demonstrated that our RT-eLAMP assay achieved similar performance to the RT-qLAMP assay, performing detection in 5–13.5 min for DENV-1 and 5.3–12 min for DENV-2. Fourth, all other requirements to the LoC cartridge and the RT-qLAMP assay include data acquisition, heating and communication, have been integrated on a PCB board and packaged as the Lacewing handheld device. By carrying out this study in the UK and in Taiwan, we have shown that our method may now be applied portably without the need for additional lab equipment. Last, the smartphone application communicating to the device via Bluetooth, synchronises all results on the AWS cloud server which can be viewed in real-time. The user experience for the app is detailed in Section S3.

Leveraging innovation towards the development of digital diagnostics and integration with remote connectivity was included as part of the recommendations of the Lancet Commission on Diagnostics (Fleming et al., 2021). The COVID-19 pandemic has further highlighted the need to bring new timely accurate diagnostics to primary health care, and the deployment outside of the hospital comes though leveraging communications technology and mobile connectivity. Digitialising health will also lead to optimised communication within the health systems i.e. patients and clinicians, and allow to rely on passive surveillance which dramatically improves current diagnostic pathways without adding any strain on the workforce. An expected workflow would be as follows. 1) The patient attends the clinic with symptoms, 2) tests are run accurately and timely, 3) results are interpreted rapidly and correctly, 4) results are reported reliably to health systems and central authority, 5) all data are collated and interpreted and 6) the interpretation translates to a public health response. Added to this workflow is the potential for data to return to the clinic with AI-based approaches to allow the implementation of clinical decision support tools which use local or regional disease incidence to enhance result interpretation.

Differentiating DENV serotypes provides essential information for outbreak surveillance. Additionally, quantifying viraemia is essential to inform disease stage and to identify a higher risk of severe dengue (Morsy et al., 2020; Vuong et al., 2020). Building upon our previous studies (Malpartida-Cardenas et al., 2019; Rodriguez-Manzano et al., 2020; Rodriguez-Manzano et al., 2021), this study demonstrates for the first time quantification with our portable LoC platform, showing statistically significant linear correlation between the RT-qLAMP and the RT-eLAMP assays. Therefore the diagnostic outcome provided by the platform matches the gold-standard results requiring laboratory equipment, trained experts and lengthy delays.

The market niche for such LoC platform lies between the ultra-low-cost and scalable NS1 antigen tests, and the lab-based PCR instrument which requires trained experts in a laboratory. As stated previously, both the cartridge and the device are designed to only include standard components which easily scale for high production volumes. The cartridge cost is expected to be dominated by the assembly stage which can be arranged locally in target countries to enable cost-efficient manufacturing. The device’s reliance on sole board fabrication and assembly means that it can be distributed at negligible costs when compared to most commercial diagnostic devices available, allowing truly scalable and deployable digital diagnostic capabilities.

When compared with reported emerging technologies for dengue detection (Table 3), it is evident that our LoC platform stands out by achieving an ultra-low LoD in a handheld format with high potential towards PoC applications. The use of label-free electrochemical sensing as opposed to an optical transduction method is key to miniaturise the instrumentation towards enabling portability, whereas many methods are reported with laboratory equipment and do not report capabilities for use in the field. The amplification chemistry also allows to achieve several orders of magnitude lower LoD than targeting proteins or detecting nucleic acid using hybridisation to surface probes which is a popular approach paired with Electrochemical Impedance Spectroscopy (EIS) or voltammetry. The complementarity of electrochemical methods and cloud syncing for digital diagnostic implementation is also an area where our LoC platform distinguishes itself from other technologies.

**TABLE 3:** Comparison of dengue detection tests reported in the literature (Darwish et al., 2018).

Transduction	Detection	Analyte	Limit of detection	Time	Handheld	Ref
Mechanism	Technique	(Target)	(LoD)	[min]		
Piezoelectric	QCM on immunochip	E protein	1.727 μg/ml	—	—	—
		NS1	0.740 g/ml	—	N	Wu et al. (2005)
		cDNA	2 PFU/ml	—	N	Chen et al. (2009)
Optical	Waveguides	NS1	5.73 pg/mm^2^	—	N	Wong et al. (2016)
	Optical fiber	E proteins	12 p.m.	15	N	Mustapha Kamil et al. (2018)
	Plasmon resonance	Viral particle	2 × 10^4^ particles/mL	—	N	Loureiro et al. (2017)
	Reflectance	DNA	0.2 a.m.	90	N	Ariffin et al. (2018)
	Fluorescence	DNA	9.4 fM	—	N	Chowdhury et al. (2018)
	ECL	RNA	25 PFU/ml	35	N	Wu et al. (2001)
	Reflectance	RNA	1 zM	30	N	Mazlan et al. (2017)
	Turbidity	RNA	10 copies/reaction	45	N	Lau et al. (2015)
Electrochemical	EIS	NS1	30 ng/ml	—	N	Cecchetto et al. (2015)
	EIS	NS1	5 ng/ml	—	N	Darwish et al. (2015)
	EIS	NS1	0.5 ng/ml	20	Y	Sinawang et al. (2016)
	EIS	DNA/RNA	1 fM	—	N	Jin et al. (2016)
	EIS & Voltammetry	RNA	3.09 nM	—	N	Oliveira et al. (2015)
	EIS & SiNWs	RNA	1.63 p.m.	—	N	Rashid et al. (2015)
	Voltammetry	RNA	17 nM	—	N	Odeh et al. (2017)
	EIS	RNA	9.55 p.m.	—	N	Rai et al. (2012)
	Potentiometry	RNA	10 fM	30	N	Zhang et al. (2010)
	Voltammetry	DNA	43 µM	—	N	Singhal et al. (2017)
	Potentiometry	RNA	1.66 aM	15	Y	This work

*QCM, quartz crystal microbalance; ECL, ElectroChemiLuminescence, EIS, Electrochemical Impedance Spectroscopy.

SiNWs, Silicon NanoWires.

We envision our system to be paired with a commercially available sample processing kit for RNA extraction, enabling sample-to-result molecular testing in the field. While we have demonstrated RT-eLAMP with two dengue serotypes, we expect that the assay (Hu et al., 2015) for DENV-3 and DENV-4 can be translated in the same manner such that all serotypes may be tested on the device. Furthermore, advances in microfluidics will allow to achieve multiplexing of several serotypes on the same test.

## 4 Conclusion

We have developed a smartphone- and cloud-connected LoC platform leveraging label-free potentiometric sensing paired with an established RT-LAMP assay for rapid detection of DENV-1 and DENV-2. The performance of our RT-eLAMP assay was validated against the gold-standard RT-qLAMP in laboratory conditions and demonstrated high correlation, rapid result times and quantification capabilities. The LoD of our RT-LAMP assay was shown to be 10 copies/reaction which is equivalent to 1.66 a.m. A study run in Taiwan demonstrated that Lacewing achieved detection of 9 DENV-2 clinical samples within 2.9–9.33 min. The platform has a great potential for PoC DENV diagnostics, offering a handheld, accurate, rapid, low-cost and connected solution ideal for low-resource settings with capabilities for disease surveillance.

The main limitation of the current platform is the lack of sample extraction capabilities, hence requiring the use of a commercially available kit to enable a sample-to-result workflow. The next step to address these is to develop an integrated sample preparation module which will deliver the extracted RNA into the reaction chamber. Additionally, the multiplexing capabilities of the current cartridge are limited to one genetic target at a time. The development of a microfluidic manifold in future work will allow to form a minimum of four reaction chambers at the surface of the chip, enabling concurrent detection of a multiplex panel with the four dengue serotypes.

Along with these future improvements, we expect that this approach will enable prompt diagnosis, accurate clinical risk-stratification of patients, and real-time surveillance of dengue epidemics. The next generation of this platform will focus on the translation and validation of an integrated sample-to-result workflow into the field.

## Data Availability

The original contributions presented in the study are included in the article/Supplementary Material, further inquiries can be directed to the corresponding author.
