# Supplementary material for: Quantitative detection of dengue serotypes using a smartphone-connected handheld lab-on-chip platform
Source: Front Bioeng Biotechnol. 2022 Sep 15;10:892853. doi: 10.3389/fbioe.2022.892853 (PMC9521504; doi:10.3389/fbioe.2022.892853)
Supplement: Supplementary file 1 [file DataSheet1.pdf]

Supporting information

Nicolas Moser [1,†,*], Ling-Shan Yu [2,†], Jesus Rodriguez-Manzano [1,5], Kenny Malpartida-Cardenas [1], Anselm Au [1], Paul Arkell [5], Chiara Cicatiello [1], Ahmad Moniri [1], Luca Miglietta [1,5], Wen-Hung Wang [3], Sheng-Fan Wang [4], Alison Holmes [5], Yen-Hsu Chen [3], and Pantelis Georgiou [1]

[1] *Centre for Bio-Inspired Technology, Department of Electrical and Electronic Engineering, Faculty of Engineering, Imperial College London, UK*
[2] *Institute of Biopharmaceutical Sciences, National Sun Yat-Sen University, Kaohsiung, Taiwan* [3] *Department of Infectious Diseases, Kaohsiung Medical University Hospital, Taiwan* [4] *Center for Tropical Medicine and Infectious Disease Research, Kaohsiung Medical University, Taiwan* [5] *NIHR Health Protection Research Unit in Healthcare Associated Infections and Antimicrobial Resistance, Department of Infectious Disease, Faculty of Medicine, Imperial College London, UK*

Correspondence*:
n.moser@imperial.ac.uk

[†] These authors have contributed equally to this work.

S1 CURRENT DIAGNOSTIC METHODS

Current methods for dengue diagnostics include the following. 1) Viral isolation is performed by culturing a clinical sample in live cells. Although this may produce definitive evidence of DENV infection (and serotyping can subsequently be performed), it is technically challenging, takes many days, and has therefore been superseded by other techniques for routine diagnosis. 2) Serological assays detect host antibodies (usually IgM and/or IgG) in clinical samples. Hemagglutination inhibition assays and enzyme-linked immunosorbent assays (ELISAs) are most commonly used, including several which are commercially available (Peeling et al., 2010). They are typically insensitive if testing is performed too early in the disease course, cannot differentiate dengue serotypes and can be non-specific due to cross-reactivity to other flaviviruses (such as the Zika virus) (Musso and Desprès, 2020). 3) Non-structural protein 1 (NS1) are present in blood or serum from the time of symptom-onset in primary dengue, and can be detected using antigen capture assays. However, quantitative NS1 assays are not currently in routine use (Young et al., 2000; Alcon et al., 2002) and are limited for use with secondary DENV infection as NS1 detection can be affected by host antibodies (Blacksell et al., 2008; Koraka et al., 2003). 4) Lateral flow assays have been developed for detection of DENV IgM, IgG and/or NS1 protein from serum, and blood samples, including capillary blood. These are portable, do not require specific training and can be produced at low cost. However, their sensitivity is variable in the diagnosis of secondary dengue, and can vary according to the infecting DENV serotype (Pang et al., 2017). 5) Nucleic acid amplification tests (NAATs) detect DENV RNA which is present in clinical samples from the time of symptom-onset in both primary and secondary dengue. These are highly sensitive and specific, and can be quantitative with higher levels of viraemia associated with more severe disease (Morsy et al., 2020). NAATs are therefore the gold-standard technique in diagnosis of dengue in the early

phase of infection. However, most commercial assays do not differentiate DENV serotypes, and requirements for laboratory infrastructure and technical expertise significantly limit their utility in remote settings.

S2 ASSAY SPECIFICITY

Fig. S1 illustrates the melting curve analysis for the DENV-1 and DENV-2 LAMP products. The melting points correlate to the ones obtained in silico with uMELT dna-uah.org/umelt/quartz), demonstrating specificity of the assays. We used adjusted conditions: Thermodynamics: United SL (1998); Free Mg2+: 1.5 mM; Mono+1: 5 mM; DMSO: 0%; Salt Correction: SL Hicks (2004) and Resolution: 0.5^{o}C).

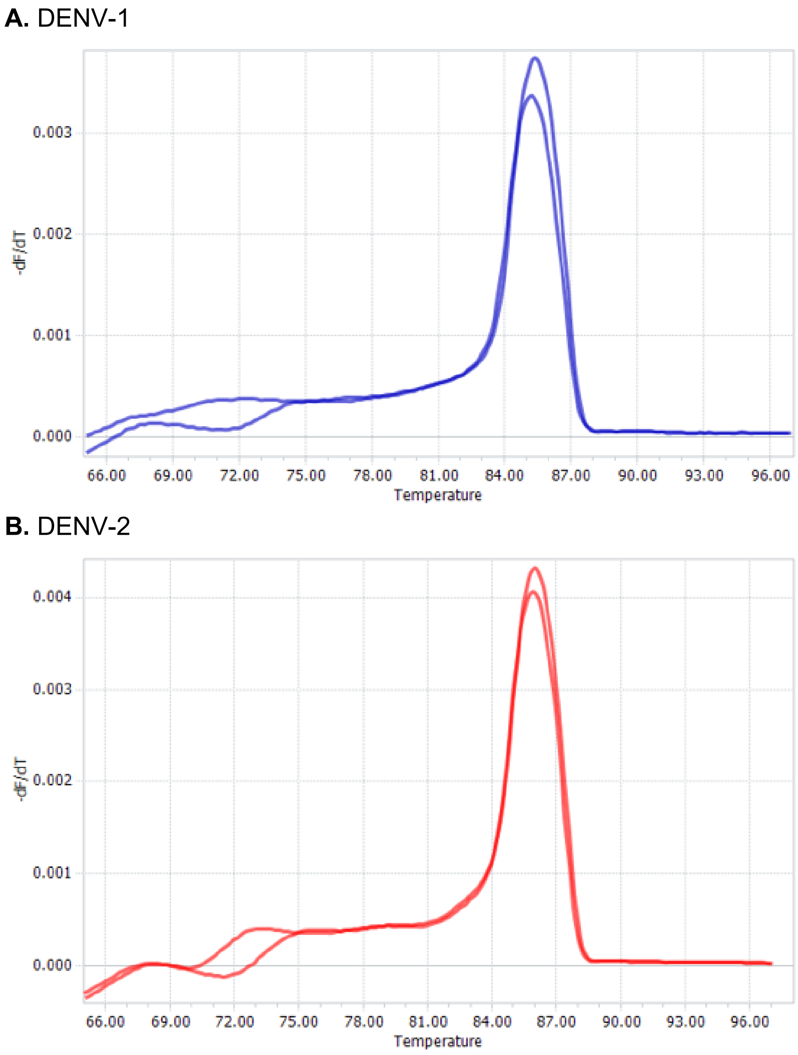

Figure S1. Melting curves of LAMP products in duplicates. The graph represents characteristic melting points for LAMP products obtained with DENV-1 and DENV-2 RNA.

S3 FIREFLY SMARTPHONE APP AND CLOUD SERVER: THE USER EXPERIENCE

The user interface for the Firefly app is depicted in Fig. S2A. On the starting screen, the user enters a new patient reference code. The app is then structured as three windows: *Information*, *Diagnostics* and *Location*. The *Information* tab prompts a summary of instructions. The *Diagnostics* tab allows to perform the test. The user has access to several actions: *Reset* initialises the CMOS microchip, *Global calibration* establishes the reference electrode voltage, *Local calibration* runs the on-chip calibration algorithm for each sensor, *Mark results* allows to specify whether a dataset is expected to be positive or negative (aimed at usage in clinical practice or research) and *Synchronise* sends the data files to the cloud server. The tab contains two main graphs: 1) a 2D image of the sensor array which shows the active area in green, and 2) an average time series from all active sensors during the reaction. The processing is performed at the end of the reaction.

The *Infection Map* tab shows the location of the real-time test reports. Fig. S2A shows that reports from synthetic samples run at Imperial College London are grouped together with indicated number of tests run. When zooming on the map, the accurate place of the reports is shown with red tags annotated for disease and location name. Fig. S2B highlights the map view with all samples presented in this study, including 18 experiments with synthetic RNA run at Imperial College London, UK, and 9 with clinical DENV-2 samples run at Kaohsiung Medical Hospital, Taiwan.

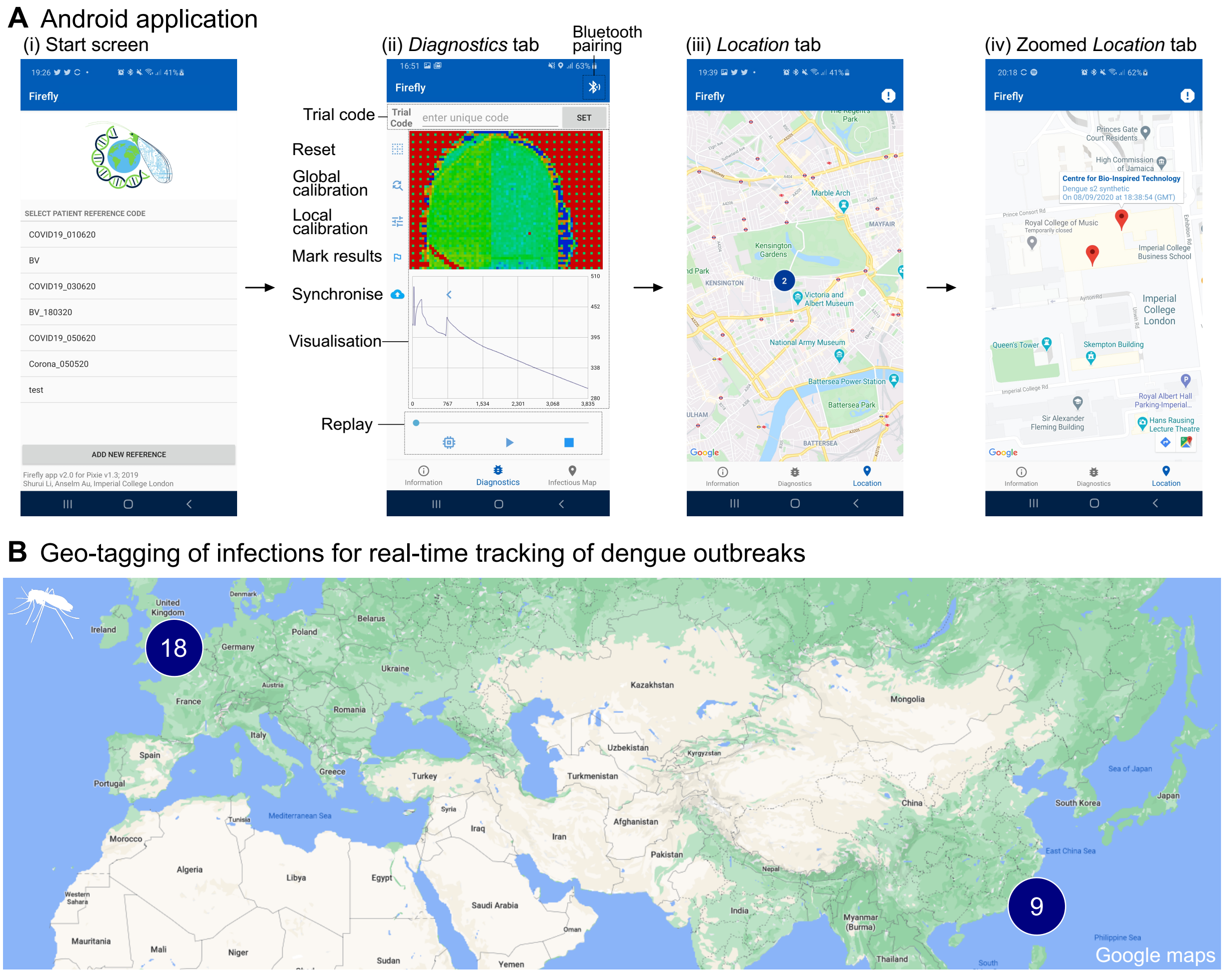

Figure S2. Screenshots of mobile software interface. A Main user screens when using the Firefly application to operate the Lacewing device, including from left to right: (i) the start screen when the user is asked to enter the patient reference code, (ii) the *Diagnostics* tab where the user can start the experiment, visualise the results in real-time and synchronise to the cloud server, (iii) the *Location* tab showing experiments run at Imperial College London and (iv) the zoomed *Location* tab showing that individual experiments are market with red pins and annotated. **B** Web interface for the infection map, showing the samples run in the UK and Taiwan in this study.

NOTES AND REFERENCES

Alcon, S., Talarmin, A., Debruyne, M., Falconar, A., Deubel, V., and Flamand, M. (2002). Enzyme-linked immunosorbent assay specific to dengue virus type 1 nonstructural protein NS1 reveals circulation of the antigen in the blood during the acute phase of disease in patients experiencing primary or secondary infections. *Journal of Clinical Microbiology* 40, 376–381. doi:10.1128/JCM.40.02.376-381.2002

Blacksell, S. D., Mammen, M. P., Thongpaseuth, S., Gibbons, R. V., Jarman, R. G., Jenjaroen, K., et al. (2008). Evaluation of the Panbio dengue virus nonstructural 1 antigen detection and immunoglobulin M antibody enzyme-linked immunosorbent assays for the diagnosis of acute dengue infections in Laos. *Diagnostic Microbiology and Infectious Disease* 60, 43–49. doi:10.1016/j.diagmicrobio.2007.07.011

Koraka, P., Burghoorn-Maas, C. P., Falconar, A., Setiati, T. E., Djamiatun, K., Groen, J., et al. (2003). Detection of immune-complex-dissociated nonstructural-1 antigen in patients with acute dengue virus infections. *Journal of Clinical Microbiology* 41, 4154–4159. doi:10.1128/JCM.41.9.4154-4159.2003

Morsy, S., Hashan, M. R., Hieu, T. H., Mohammed, A. T., Elawady, S. S., Ghosh, P., et al. (2020). The association between dengue viremia kinetics and dengue severity: A systemic review and meta-analysis. *Reviews in Medical Virology* 30, 1–10. doi:10.1002/rmv.2121

Musso, D. and Desprès, P. (2020). Serological diagnosis of flavivirus-associated human infections. *Diagnostics* 10, 1–13. doi:10.3390/diagnostics10050302

Pang, J., Chia, P. Y., Lye, D. C., and Leo, Y. S. (2017). Progress and Challenges towards Point-of-Care Diagnostic Development for Dengue. *Journal of Clinical Microbiology* 55, 3339–3349. doi:10.1128/JCM.00707-17

Peeling, R. W., Artsob, H., Pelegrino, J. L., Buchy, P., Cardosa, M. J., Devi, S., et al. (2010). Evaluation of diagnostic tests: dengue. *Nature Reviews Microbiology* , S30–S37doi:10.1038/nrmicro2459

Young, P. R., Hilditch, P. A., Bletchly, C., and Halloran, W. (2000). An antigen capture enzyme-linked immunosorbent assay reveals high levels of the dengue virus protein NS1 in the sera of infected patients. *Journal of Clinical Microbiology* 38, 1053–1057. doi:10.1128/jcm.38.3.1053-1057.2000
